# Major benefits of guarding behavior in subsocial bees: implications for social evolution

**DOI:** 10.1002/ece3.2387

**Published:** 2016-09-01

**Authors:** Michael Mikát, Kateřina Černá, Jakub Straka

**Affiliations:** ^1^ Department of Zoology Faculty of Science Charles University in Prague Praha Czech Republic

**Keywords:** Apidae, *Ceratina*, mass provisioning, maternal care, nesting strategy, offspring protection

## Abstract

Parental care is a behavior that increases the growth and survival of offspring, often at a cost to the parents' own survival and/or future reproduction. In this study, we focused on nest guarding, which is one of the most important types of extended parental care; we studied this behavior in two solitary bee species of the genus *Ceratina* with social ancestors. We performed the experiment of removing the laying female, who usually guards the nest after completing its provisioning, to test the effects of nest guarding on the offspring survival and nest fate. By dissecting natural nests, we found that *Ceratina cucurbitina* females always guarded their offspring until the offspring reached adulthood. In addition, the females of this species were able to crawl across the nest partitions and inspect the offspring in the brood cells. In contrast, several *Ceratina chalybea* females guarded their nests until the offspring reached adulthood, but others closed the nest entrance with a plug and deserted the nest. Nests with a low number of provisioned cells were more likely to be plugged and abandoned than nests with a higher number of cells. The female removal experiment had a significantly negative effect on offspring survival in both species. These nests frequently failed due to the attacks of natural enemies (e.g., ants, chalcidoid wasps, and other competing *Ceratina* bees). Increased offspring survival is the most important benefit of the guarding strategy. The abandonment of a potentially unsuccessful brood might constitute a benefit of the nest plugging behavior. The facultative nest desertion strategy is a derived behavior in the studied bees and constitutes an example of an evolutionary reduction in the extent of parental care.

## Introduction

Parental care is a diverse life history trait that includes various types of behaviors (Tallamy and Wood 1986; Trumbo 2012) and that influences other life history characteristics of animals (Gilbert and Manica 2010). The three primary types of parental care are provisioning with food, offspring guarding, and building nests or shelters (Wilson 1971; Thiel 2000; Smiseth et al. 2012).

Parental care is crucial for offspring survival in certain species (Smiseth et al. 2012), while it only increases the fitness of the offspring in others (Martins et al. 1998; Mas and Kölliker 2008). Offspring are usually more dependent if the main form of care is provisioning rather than guarding (Smiseth et al. 2012). The dependency of the offspring on parental care is also affected by the age of the offspring, that is, the younger stages are more parent dependant (Coville and Griswold 1984; Eggert et al. 1998; Smiseth et al. 2003).

One of the most important features that affect the life history of species with respect to parental care is the trade‐off between the length of care for the offspring and the number of offspring produced in a lifetime. Longer parental care causes a decrease in the number of offspring over time (Gross 2005; Smiseth et al. 2012; Kölliker et al. 2015). Similarly, when offspring are produced in separate clutches, the parents must decide whether it is more effective to care for the current clutch or to desert that clutch and establish a new one (Olmstead and Wood 1990; Mas and Kölliker 2008).

The aculeate Hymenoptera are one of the most important and most studied groups of invertebrates with parental care (Wilson 1971; Tallamy and Wood 1986; Linksvayer and Wade 2005). In nonkleptoparasitic aculeate Hymenoptera, the parental care typically consists of nest building and provisioning accompanied with nest guarding in certain species (Wilson 1971; Michener 1974). The offspring are provisioned in two primary modes in the aculeate Hymenoptera. The mass provisioners collect and supply a cell with all the necessary food for the young; then, they deposit an egg and close the cell. Typically, the adults do not interact with the offspring; therefore, guarding is not likely to be important for offspring survival (Strohm and Linsenmair 2000; Field 2005). In contrast, the progressive provisioners feed (and guard) their offspring regularly throughout larval development (Field and Brace 2004; Field 2005). Interestingly, there are a few exceptions to this scheme. For example, mothers stay with their offspring until they reach adulthood in certain dung beetles (Trumbo 2012), in eusocial mass‐provisioning halictid bees (Michener 1974) and in solitary populations of *Halictus rubicundus* (Eickwort et al. 1996). A remarkable strategy of parental care is also found in the mass‐provisioning genus *Ceratina* (Sakagami and Maeta 1977; Rehan and Richards 2010a).

Bees of the cosmopolitan genus *Ceratina* nest in dead stems or sticks with pith (Hogendoorn and Velthuis 1999; Michener 2007) and are either solitary or facultatively eusocial (Sakagami and Maeta 1977; Rehan et al. 2009). The ancestor of this genus (and of the entire Xylocopinae subfamily) was facultatively eusocial (Rehan et al. 2012). *Ceratina* bees guard their nests after the end of provisioning. The nest is guarded by either a single female (mother) or more than one adult female (mother and daughter) until the offspring reach maturity (Sakagami and Laroca 1971; Rehan and Richards 2010a). This guarding behavior is found in all the studied species of *Ceratina* bees (Rehan et al. 2010b). In addition, the guarding behavior is not only a passive strategy in *Ceratina* bees. Females are subsocial and typically crawl through the cell partitions in the nest to examine the offspring (Maeta et al. 1997; Rehan and Richards 2010a).

To test the importance of nest guarding, we performed an experiment where the guarding individual was removed from a nest, and the fate of the offspring in the unguarded nest was monitored. Removal experiments performed in certain invertebrates showed that the absence of the parents could result in an increased mortality of the offspring and a poorer growth (Eggert et al. 1998; Thiel 2000; Kölliker and Vancassel 2007; Werneck et al. 2012).

For the aculeate Hymenoptera, only a few experiments in which the guarding female(s) were removed were performed, primarily with eusocial (Smith et al. 2003) or communal species (Kukuk et al. 1998). Removal experiments were further used to test the role of males in species with biparental care (Coville and Griswold 1984) or in eusocial species with extended male care (Sen and Gadagkar 2006; Lucas and Field 2011). However, no removal experiments have been performed to test the role of the female mother in solitary aculeate Hymenoptera. Moreover, there are observational studies that compared the mortality of brood cells in guarded and naturally orphaned nests, but these studies have an insufficient number of observations and provide ambiguous results (Sakagami and Maeta 1977; Eickwort et al. 1996; Rehan et al. 2009).

The lack of relevant studies that examined the fate of orphaned nests is surprising because nest failure after orphaning is often argued to be one of the most important driving forces in the evolution of sociality. The insurance of offspring survival in the case of the death of the founder represents an important selection pressure for the maintenance of eusociality (Gadagkar 1990; Queller 1994) and for the existence of pleometrotic nest founding (Queller 1994; Queller et al. 2000). The probability of offspring survival after the death of the mother is also considered to affect the benefits obtained from the progressive provisioning (Field 2005).

**Figure 1:**
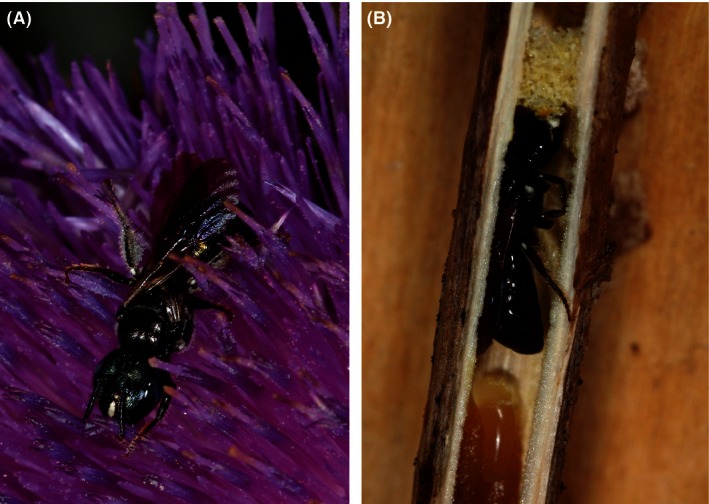
*Ceratina chalybea* collecting pollen from *Onopordum acanthium* (A), and *Ceratina cucurbitina* inside the nest (B). Photograph Lukáš Janošík.

In this study, we tested the importance of nest guarding for mass‐provisioning *Ceratina* bees, which are solitary species with social ancestors. We demonstrated that there are two different strategies to protect the nests, which include an alternative to nest guarding that had not been previously recognized in *Ceratina* bees. We studied these different nesting strategies in two relatively distantly related species: *Ceratina cucurbitina* (Rossi) and *Ceratina chalybea* Chevrier (Fig. 1; Rehan et al. 2010a; Terzo 1998).

## Materials and Methods

### Study site

The observations and experiments were conducted in the Podyjí National Park, near Znojmo, in the Czech Republic. We received permission to perform the experiments in the National Park (NPP 0781/2011). Most of the data were collected in the locality of the Havranické vřesoviště heathland (48°48′33.595″N, 15°59′35.149″E), but additional data were collected at the Šobes locality (48°49′0.124″N, 15°58′37.708″E). The localities were both open grassy habitats, with solitary trees and shrubs. The study was performed between 2012 and 2014, from the end of May to the beginning of August. The studied species (*C. cucurbitina* and *C. chalybea*) are abundant in the studied localities. They naturally nest in broken twigs and stems of *Rosa canina*,* Centaurea* spp., *Linaria genistifolia*,* Verbascum* spp., and other plants with pith.

### Preparation of nesting opportunities

All the experiments were performed on nests that were artificially prepared as nesting opportunities. Sheaves consisting of 20 cut plant stems were distributed in the study site. Each stem was approximately 30–50 cm long, with an inner pith of more than 3 mm wide in the upper end (Fig. S1). We used stems of *Solidago* spp., *Helianthus tuberosus*,* Echinops sphaerocephalus,* and *Dipsacus sylvestris*. The sheaves were fixed to a rod for stabilization, and they were then fixed to the ground and placed in suitable nesting sites. These nesting opportunities were distributed in the studied localities in April before the beginning of the nesting season. We installed 2600 sheaves, which corresponded to 52,000 nesting opportunities.

### Nest dissection

All the nests selected for dissection were collected after 7 p.m. CEST. By collecting the nests early in the evening, we ensured that all the inhabitants had returned to the nest and were inside because *Ceratina* bees are not active at this time (Herrera 1990). The collected nests were carefully opened with a knife in the field laboratory. The original nest structure and the following parameters were recorded in all the analyzed nests: stem substrate species, presence of guarding adult female, the presence and number of living offspring, and the presence of natural enemies. We considered nests as attacked by a focal enemy when we observed such enemy species in the nest at the time of dissection. In the case of nest usurpation, we marked those nests as usurped when discarded pollen or a low number of fresh cells with eggs were observed in the nest.

### Description of the guarding strategy

This study was performed in 2012 and 2013. We selected approximately 30 sheaves and dissected all the nests in them every 7–9 days from mid‐June to mid‐August. In total, we selected 360 sheaves for this experiment; however, not all sheaves contained nests. All the nests from the selected sheaves were dissected. Additional natural nests were collected in 2012; the substrates were *Rubus* spp., *Artemisia* spp., and *Helianthus tuberosus*.

Only nests with full brood and an undisturbed inner structure were used for description of guarding strategy. Nests that contained a larva or a pupa in the outermost cell in the line were considered as full brood nests (Rehan and Richards 2010a,b; Vickruck et al. 2011).

We recorded the presence and position of the guarding female.

We used 60 nests of *C. cucurbitina* (26 in 2012 and 34 in 2013) and 201 nests of *C. chalybea* (14 in 2012 and 187 in 2013) for description of guarding strategy of species.

### Comparison of guarding strategies of *C. chalybea*

In *C. chalybea*, we discovered two alternative guarding strategies – nest guarding versus nest abandonment. For comparison of these strategies, we used only data from 2013 (187 nests from 114 sheaves). To compare these strategies, we recorded presence of guarding female, architecture of last brood cell (open vs. closed), length of nest, number of brood cells, number of brood cells with living offspring, and number of brood cells with parasitized offspring.

### Phenology of *C. chalybea*

The analysis of the phenology was performed in 2013. For this analysis, 108 sheaves were selected, but only 74 contained *C. chalybea* nests. Each sheaf was regularly controlled between 10 May 2013 and 25 July 2013. The sheaves were controlled every 1–3 days. New nests and the content of older nests were recorded using a pocket flashlight during each control. Confirmed by a later nest dissection, we could reliably observe and determine the content of the first 4 or 5 cm of depth in the nests. The founding date of a *C. chalybea* nest was stated as either a) the date of the first observation of a new nest, where the presence of *C. chalybea* was subsequently recorded, or b) the date of the first observation of a *C. chalybea* female in a nest where another species of insect was the owner of the nest before a *C. chalybea* female was regularly observed. The date of nest plugging was the first date when a plug was observed and the female disappeared. In total, we used 133 observed nests in this analysis, of which 44 were plugged nests.

### Removal of the guarding female

This experiment was performed in the nesting seasons of 2013 and 2014 (from the end of June to the beginning of August). The nests and sheaves used for this experiment were different from the nests used for the analysis of the guarding strategy. For this experiment, we selected 500 sheaves in 2013 and 700 sheaves in 2014 for the analysis, but only in some sheaves, we found nests in appropriate stage.

Nests of *C. cucurbitina* or *C. chalybea* with a guarding female at the nest entrance were selected. The guarding female was pulled from the nest. The nest was visually inspected by shining a light inside the nest entrance, and only completely provisioned nests were used for the experiment. A completely provisioned nest of *C. chalybea* was characterized by either a visible partition or the presence of pollen or a larva at the nest entrance. A completely provisioned nest of *C. cucurbitina* was distinguished by a visible partition near the nest entrance.

When a nest was appropriate for this experiment, the guarding female was either removed (two‐thirds of the cases) or was returned to the nest to serve as a control (one‐third of the cases). To evaluate the influence of nest guarding on the nest success, we performed gradual dissections of all the experimental nests. The dissections were performed at 3, 8, or 20 days after the treatment, using one‐third of the nests in each interval. Based on our preliminary results, the interval between the end of provisioning and the emergence of the first adult juveniles took approximately 20 days, which we considered as the approximate duration for the complete provisioning of nests.

The female was removed in 140 nests of *C. chalybea* (72 in 2013 and 68 in 2014) and 208 nests of *C. cucurbitina* (123 in 2013 and 85 in 2014). We used 72 nests of *C. chalybea* (35 in 2013 and 37 in 2014) and 108 nests of *C. cucurbitina* (62 in 2013 and 46 in 2014) as controls. In total, 212 nests from 174 sheaves in *C. chalybea* and 315 nests from 210 sheaves in *C. cucurbitina* were used.

For comparison of mortality in outermost and second outermost cells, we used only nests in which these cells were preserved (nest partitions were not disturbed). Therefore, we used only subset of nests for this analysis: 155 nest from 134 sheaves for *C. chalybea* and 150 nest form 124 sheaves in *C. cucurbitina*.

### Comparison between unguarded *C. chalybea* nests and nests with the female removed

We performed an analysis for evaluating the proportion of dead offspring in unguarded *C. chalybea* nests to decide whether it was the cause or the consequence of nest abandonment by the female. For this analysis, we used nests of *C. chalybea* with undisturbed structure from 2013 (50 nests from 45 sheaves) and unguarded full brood nests, which we observed and for which we noted a plug date. The nests were dissected within 30 days from plugging (22 nests from 16 sheaves).

### Data analyses

The statistical analyses were conducted in the program R 3.1.0 (R Development Core Team, 2011). When we have more nests from one sheaf, we used mean values per sheaf in all linear models or generalized linear models.

We performed these analyses:

1. Association between guarding and nest architecture of *C. chalybea*: chi‐square test.
2. Comparison of nests features between guarded and unguarded *C. chalybea* nests. Explanatory variable was in all cases presence of guarding female. We performed five analyses for different dependent variables. We used linear model for length of nest and length of nests entrance, poison generalized linear model for number of provisioned cells, and number of live offspring and binomial generalized linear model for proportion of cells parasited by chalcidoid wasps. Model equation: response ˜ guarding strategy.
3. Comparison of nest founding date between guarded and unguarded *C. chalybea* nests: Binomial generalized linear model. We used guarding as dependent variable and date of nest founding as explanatory variable. Model equation: guarding strategy ˜ date, family = binomial.
4. Influence of female removal to nest fate: We used binomial generalized linear model. We tested these explanatory variables: year, difference between data of removing and dissecting, treatment (removing of female vs. control), and all double interactions. We performed analyses for these dependent variables: at least one live offspring in nest, the presence of ants in nest, the presence of chalcidoid wasp, nest usurpation, live offspring in outermost cell, and live offspring in second outermost cell. Model equation: response ˜ (year + date difference + treatment)^2, family = binomial.
5. Comparison of nest features between nests with removed female and plugged nests: We used binomial generalized linear model. We used these explanatory variables: time of nests abandonment and treatment (removing vs. control). We tested these response variables: proportion of live offspring and proportion of chalcidoid wasps. Model equation: response ˜ date difference*treatment, family = binomial

## Results

### Guarding strategy of *C. cucurbitina*

Full brood nests of *C. cucurbitina* were guarded by the mothers in 58 of the 60 examined nests (96.6%). The cell partitions in the nest of this species were relatively more fragile than those in the nests of *C. chalybea*. The adult females were occasionally observed inside the nests around the brood cells as if they were inspecting them (5.1% of the nests with the owner female, 6 of 117 nests). This behavior agrees with the observation that excrements typically accumulate at the bottom of the nest. Moreover, no case of social nesting and male presence in the nests was recorded; however, we observed females feeding adult offspring with pollen.

### Guarding strategy of *C. chalybea*

**Figure 2:**
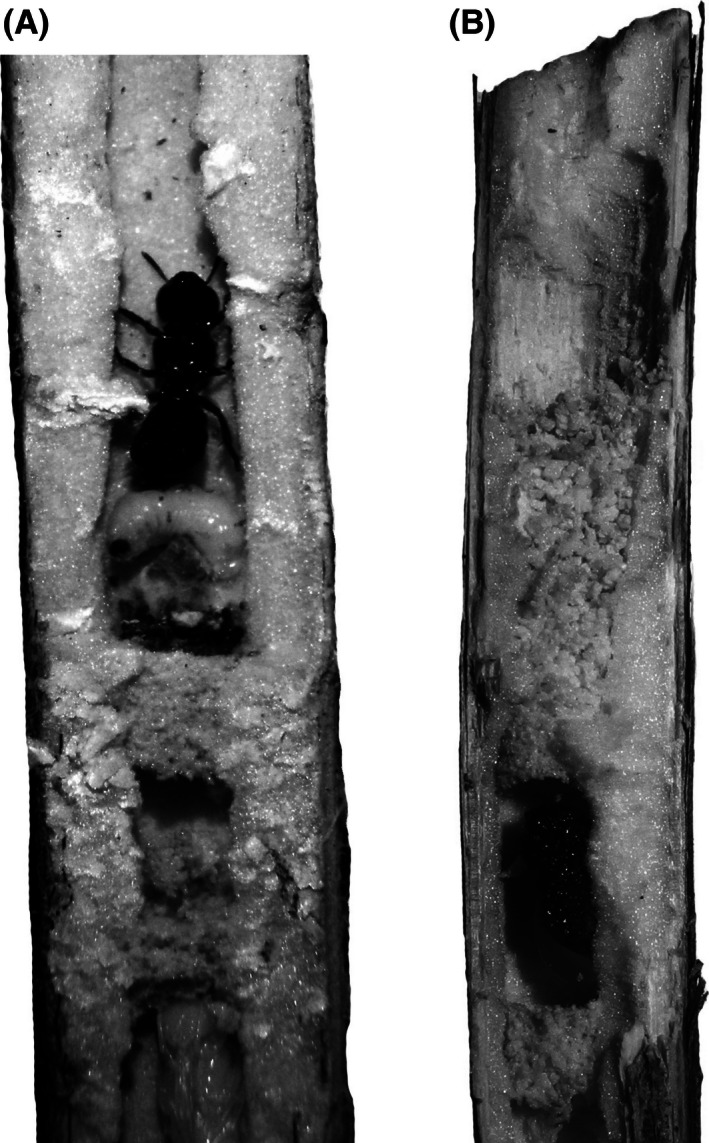
Comparison of the nest architecture of guarded (A) and plugged (B) *Ceratina chalybea* nests.

*Ceratina chalybea* had two alternative types of nest protection (Fig. 2, Table 1). The nest was either guarded by females sitting by the outermost cell or was left unguarded and closed with a plug formed with pith fillings. This nest plug was similar to the material used for the nest partitions, but it was thicker and was approximately 1.5 cm in length. A strong association between the architecture of the outermost cell and the presence of a guarding mother was detected (chi‐square test, χ^2^ = 56.8228, df = 1, *P* < 0.0001, Table 1). These differences in nest architecture did not reflect the stage of ontogeny of the nests. All the stages of offspring (larva, pupa, or young adult) were found in the outermost cells in both types of architectures. Therefore, the nest plugging represented an alternative guarding strategy in this species.

**Table 1:** Characteristics of guarded and plugged *Ceratina chalybea* nests (season 2013)

	Unguarded nests	Guarded nests	Together
Total number of nests	75	112	187
Number of nests with an unclosed outermost cell	2	106	108
Number of plugged nests	73	6	79
Chi‐square test	χ^2^ = 56.8228. df = 1. *P* < 0.0001		
Length of nest (cm)			
Mean	19.18	23.51	21.77
Maximum	31.4	34.7	34.7
Minimum	5.9	8.2	5.9
Standard deviation	5.21	5.66	5.87
Linear model	*F* = 24.26, df = 1, *P* < 0.0001		
Length of the nest entrance (cm)			
Mean	2.81	4.33	3.72
Maximum	17.8	14.6	17.8
Minimum	0.5	1.0	0.5
Standard deviation	2.50	2.15	2.41
Linear model	*F* = 17.531, df = 1, *P* < 0.0001		
Number of provisioned cells			
Mean	4.33	6.79	5.80
Maximum	8	11	11
Minimum	1	2	1
Standard deviation	1.56	2.11	2.25
Poisson's GLM family	Deviance = 21.828, residual deviance = 55.391, df = 1 *P* < 0.0001		
Number of cells with live offspring			
Mean	1.29	4.73	3.35
Maximum	8	11	11
Minimum	0	1	0
Standard deviation	1.69	2.410	2.73
Poisson's GLM family	Deviance = 95.487, residual deviance = 143.85, df = 1, *P* < 0.0001		
Proportion of parasitized cells by Chalcidoid wasps			
Mean	0.10	0.07	0.08
Maximum	1.00	0.88	1.00
Minimum	0.00	0.00	0.00
Standard deviation	0.23	0.16	0.19
binomial GLM family	Deviance = 0.0001, residual deviance = 30.9, *P* = 0.9928		

A mother of *C. chalybea* was never observed inside the nest beyond the outermost cell partition. Excrements of larvae remain inside the brood cells, and they are not removed to the bottom of the nest. The cell partitions remained undisturbed until the offspring matured and were more compact and thicker than the partitions of the *C. cucurbitina* nests. No case of social nesting was recorded, but a male was found in the nest entrance of full brood nests in a few cases (3%, 6 nests of 201).

### Differences between guarded and unguarded nests of *C. chalybea*

**Figure 3:**
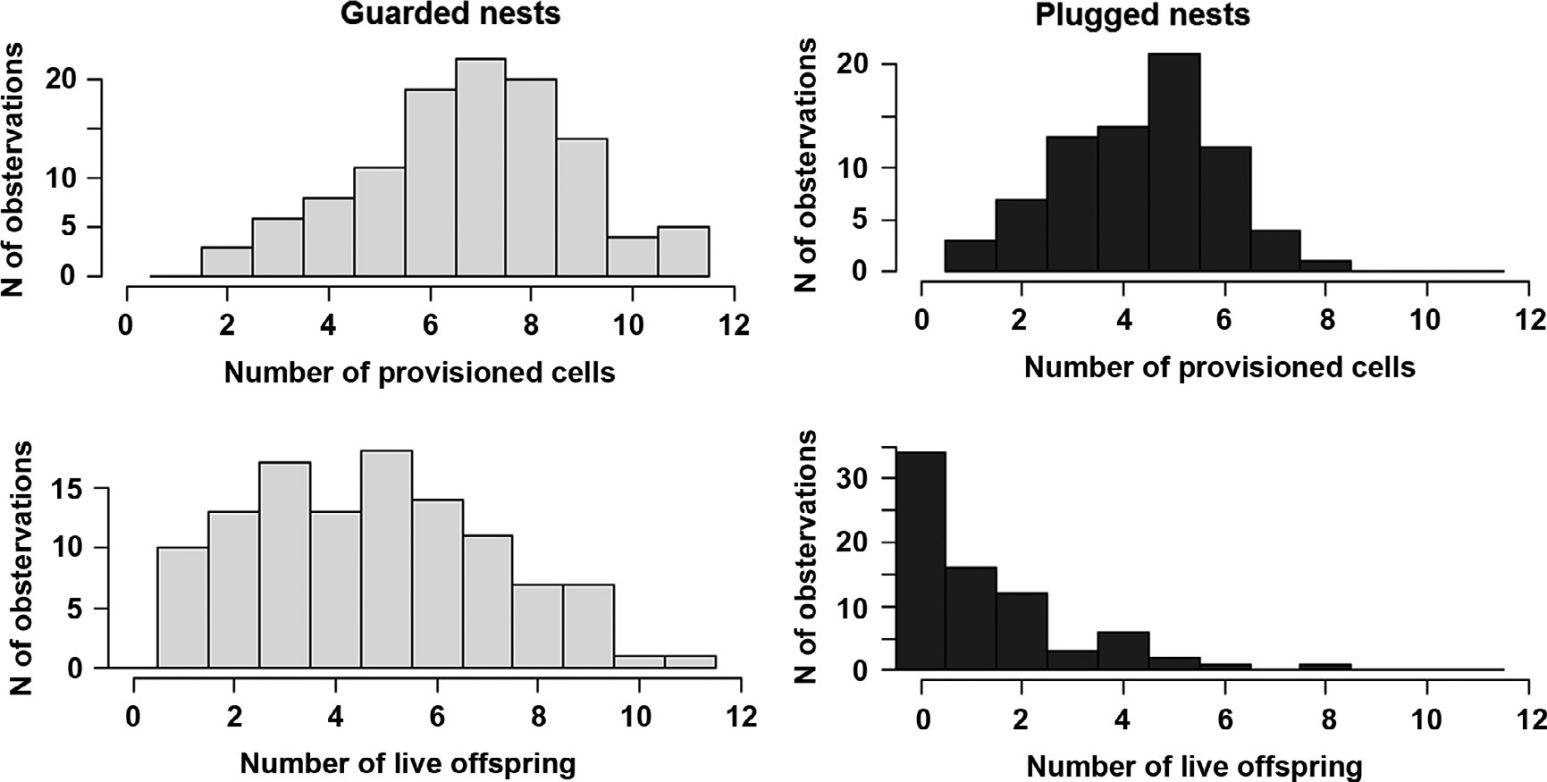
Comparison of guarded and plugged *Ceratina chalybea* nests with regard to the number of provisioned cells and the number of live offspring. Light gray bars represent guarded nests; dark gray bars represent plugged nests.

Guarded nests are more common than unguarded ones (112 guarded nests of 187 nests). Several important characteristics differentiated the guarded and plugged nests (Table 1). For example, the guarded nests were significantly longer (linear model, *F* = 24.26, df = 1, *P* < 0.0001; Table 1) and had a higher number of provisioned cells (Poisson's GLM, deviance = 21.83, df = 1, *P* < 0.0001; Table 1, Fig. 3) than the plugged nests. The guarded nests also had a higher number of live offspring (Poisson's GLM, deviance = 95.45, df = 1, *P* < 0.0001; Table 1, Fig. 3). On the other hand, there is no significant difference in proportion of cells parasitized by chalcidoid wasps (binomial GLM, deviance = 0.0001, df = 1, *P* = 0.993).

### Phenology of nest founding and plugging in *C. chalybea*

*Ceratina chalybea* founded new nests predominantly between May and mid‐June; however, a lower frequency of nest founding was observed in the second part of June and throughout July (Fig. S2). The nest plugging began by mid‐June and had the maximum occurrence between June 20 and 23. The plugging continued through the last week of June and throughout July with a lower frequency. After mid‐June, the numbers of newly founded and plugged nests were similar (Fig. S2).

There was no significant difference between guarded and plugged nest in date of nest founding (binomial GLM, deviance = 0.2226, df = 1, *P* = 0.6371). However, late‐founded nest (after June 20) were plugged very rarely.

### Effect of removal of the guarding female

**Figure 4:**
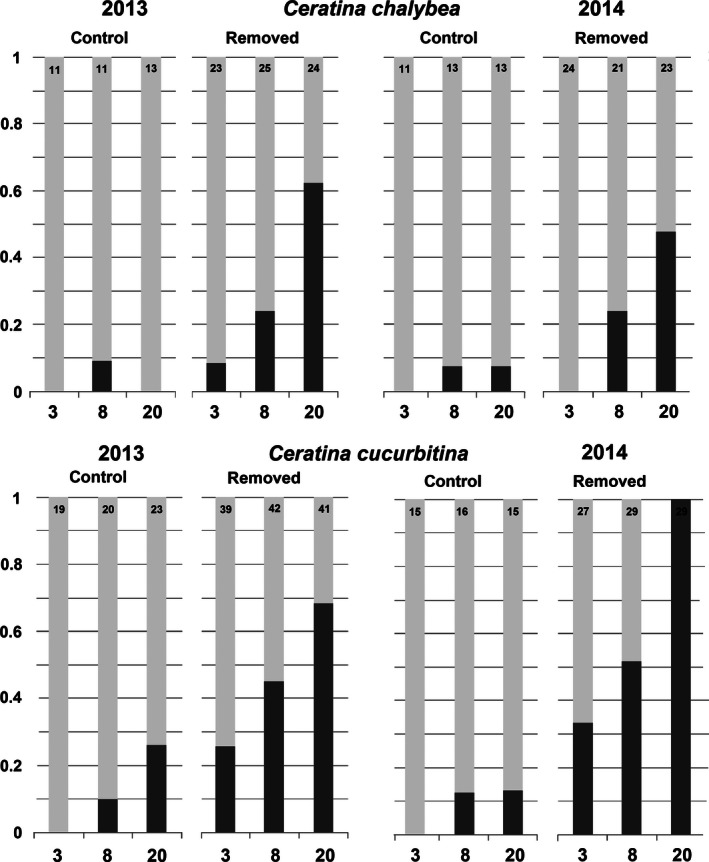
Proportion of survival of control nests and nests with the female removed of *Ceratina chalybea* and *Ceratina cucurbitina*. Light gray columns represent nests with at least one surviving offspring; dark gray columns represent nests with no surviving offspring. The numbers below the columns represent the number of days since the beginning of the treatment to the nest dissection. The numbers on the top of the columns represent the number of nests examined.

When the guarding female was removed, the decrease in offspring survival was highly significant in both species (binomial GLM, *C. cucurbitina*: deviance = 39.18, df = 1, *P* < 0.0001, *C. chalybea*: deviance = 10.92, df = 1, *P* = 0.0009; Table 2 and Fig. 4).

**Table 2:** Effects of the removal of the guarding female on nest survival and natural enemy occurrence. This table shows the results of a binomial GLM. The interaction among factors is marked by “*.” Significant effects are in bold

Variable	df	Deviance	Residual Df	Residual deviance	*P*‐value
*Ceratina cucurbitina*, dependent variable: at least one live offspring					
NULL			209	229.79	
Year	1	0.16	208	229.63	0.6855
Date difference	1	22.14	207	207.49	**<0.0001**
Treatment	1	39.18	206	168.31	**<0.0001**
Year *date difference	1	0.79	205	167.52	0.3736
Year*treatment	1	1.70	204	166.45	0.3012
Date difference*treatment	1	0.14	203	166.31	0.7081
*Ceratina cucurbitina*, dependent variable: presence of chalcidoid wasps					
NULL			209	154.00	
Year	1	0.19	208	153.81	0.6669
Date difference	1	2.03	207	151.28	0.1116
Treatment	1	10.53	206	140.85	**0.0012**
Year*date difference	1	0.63	205	140.22	0.4258
Year*treatment	1	3.66	204	136.94	0.0703
Date difference*treatment	1	2.60	203	134.51	0.1193
*C. cucurbitina*, dependent variable: presence of ants					
NULL			209	120.64	
Year	1	0.99	208	119.65	0.3200
Date difference	1	2.50	207	117.00	0.1039
Treatment	1	11.30	206	105.97	**0.0009**
Year*date difference	1	1.10	205	104.96	0.3148
Year*treatment	1	0.00	204	104.96	0.9895
Date difference*treatment	1	0.14	203	104.82	0.7112
*C. cucurbitina*, dependent variable: nest usurped					
NULL			209	77.11	
Year	1	0.46	208	76.65	0.4964
Date difference	1	0.03	207	76.62	0.8741
Treatment	1	1.20	206	75.43	0.2741
Year*date difference	1	2.98	205	72.44	0.0842
Year*treatment	1	0.93	204	71.51	0.3341
Date difference*treatment	1	0.05	203	71.46	0.8204
*C. cucurbitina*, dependent variable: live offspring in outermost cell					
NULL			123	122.45	
Year	1	1.54	122	120.80	0.1996
Date difference	1	5.30	121	115.78	**0.0249**
Treatment	1	4.00	120	111.77	**0.0453**
Year*date difference	1	1.69	119	110.53	0.2661
Year*treatment	1	0.75	118	109.78	0.3879
Date difference*treatment	1	5.77	117	104.13	**0.0174**
*C. cucurbitina*, dependent variable: live offspring in second outermost cell					
NULL			123	111.23	
Year	1	3.78	122	107.38	**0.0498**
Date difference	1	21.90	121	86.29	**<0.0001**
Treatment	1	12.97	120	74.06	**0.0004**
Year*date difference	1	0.70	119	73.36	0.4026
Year*treatment	1	0.42	118	72.94	0.5187
Date difference*treatment	1	2.20	117	70.64	0.1292
*C. chalybea*, dependent variable: at least one live offspring					
NULL			173	151.19	
Year	1	0.07	172	151.12	0.7962
Date difference	1	19.91	171	131.21	**<0.0001**
Treatment	1	10.92	170	120.28	**0.0009**
Year*date difference	1	0.20	169	120.08	0.6570
Year*treatment	1	0.28	168	119.81	0.5990
Date difference*treatment	1	2.22	167	117.39	0.1196
*C. chalybea*, dependent variable: presence of chalcidoid wasps					
NULL			173	152.75	
Year	1	0.14	172	152.61	0.7059
Date difference	1	8.82	171	143.74	**0.0029**
Treatment	1	0.57	170	143.18	0.4516
Year*date difference	1	1.12	169	141.22	0.1613
Year*treatment	1	0.38	168	140.83	0.5359
Date difference*treatment	1	0.42	167	140.41	0.5176
*C. chalybea*, dependent variable: presence of ants					
NULL			173	78.96	
Year	1	4.16	172	74.03	**0.0265**
Date difference	1	2.06	171	71.96	0.1503
Treatment	1	2.17	170	69.31	0.1034
Year*date difference	1	0.67	169	68.62	0.4037
Year*treatment	1	1.20	168	67.59	0.3117
Date difference*treatment	1	0.54	167	67.06	0.4640
*C. chalybea*, dependent variable: nest usurped					
NULL			173	91.15	
Year	1	2.39	172	88.70	0.1172
Date difference	1	0.65	171	88.05	0.4215
Treatment	1	6.18	170	81.88	**0.0129**
Year*date difference	1	0.04	169	81.83	0.8352
Year*treatment	1	1.86	168	80.47	0.2438
Date difference*treatment	1	0.66	167	79.82	0.4174
*C. chalybea*, dependent variable: live offspring in outermost cell					
NULL			133	137.47	
Year	1	0.02	132	137.44	0.8828
Date difference	1	0.99	131	136.49	0.3278
Treatment	1	50.62	130	85.87	**<0.0001**
Year*date difference	1	0.18	129	85.69	0.6709
Year*treatment	1	0.37	128	85.31	0.5416
Date difference*treatment	1	1.51	127	83.80	0.2185
*C. chalybea*, dependent variable: live offspring in second outermost cell					
NULL			133	120.08	
Year	1	14.54	132	105.54	**0.0001**
Date difference	1	3.13	131	101.77	0.0521
Treatment	1	1.81	130	100.42	0.2456
Year*date difference	1	1.99	129	98.59	0.1749
Year*treatment	1	0.02	128	98.55	0.8636
Date difference*treatment	1	2.35	127	96.04	0.1129

The probability of nest failure increased with the time spent without a guarding female (Fig. 4, Table 2). Twenty days after the experimental treatment, all the offspring died in 21.6% (8 of 37) of the control nests, while this percentage was 81.4% (57 from 70) in the nests where the female *C. cucurbitina* was removed. All the offspring died in 3.9% (1 of 26) of the control nests and in 53.1% (25 of 47) of the nests where the *C. chalybea* female was removed (Fig. 4).

The probability of offspring survival in the outermost cell (unclosed cell with guarding female) was significantly affected by the removal of the female in *C. chalybea* (binomial GLM, deviance = 50.62, df = 1, *P* < 0.0001, Table 2). In contrast, the mortality of the second outermost cell (standard cell without contact between mother and offspring) was not significantly affected by the female removal in *C. chalybea* nests with an undisturbed structure (binomial GLM, deviance = 1.81, df = 1 *P* = 0.2456; Table 2). Conversely, in *C. cucurbitina*, the mortality of the outermost and the second outermost cell was affected by the treatment (Table 2).

### Reasons of failure of unguarded nests

**Figure 5:**
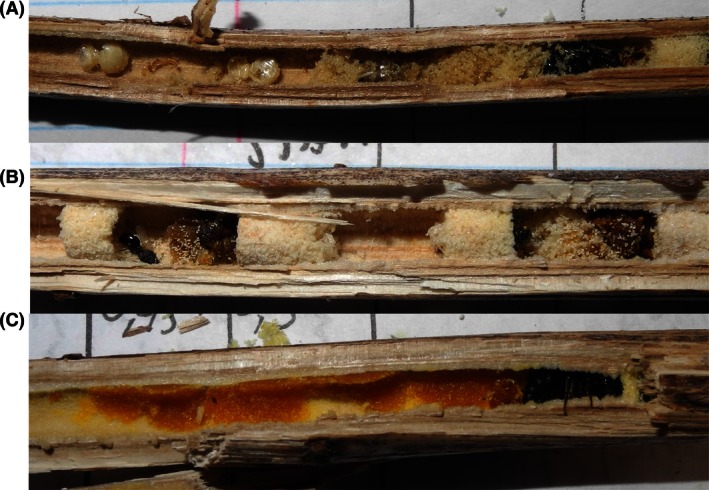
Examples of nests that were attacked by natural enemies after removal of female *Ceratina chalybea*: nest attacked by predator(s), most likely ants or Dermaptera (A), nests with cell parasitized by a chalcidoid wasp (B), and nest usurped by another *C. chalybea* female, with the offspring from the first female discarded (C).

**Figure 6:**
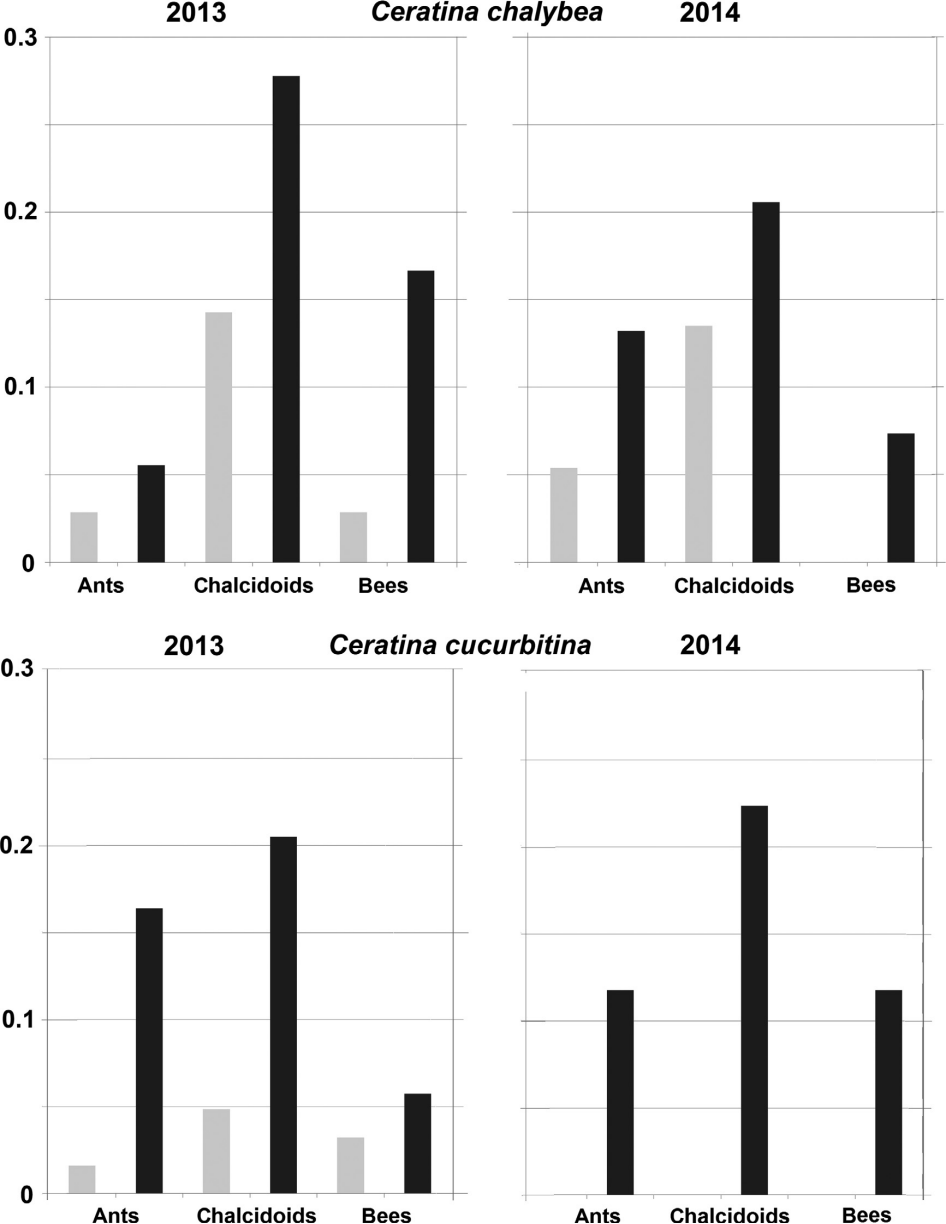
Proportion of *Ceratina chalybea* and *Ceratina cucurbitina* nests attacked by natural enemies. Light gray columns represent the control nests; dark gray columns represent the nests where the female was removed.

The most important natural enemies were the chalcidoid parasitoids, predatory ants, and other bees that usurped the nests (Figs 5 and 6). In general, the occurrence of these enemies was more frequent and had a stronger effect on *C. cucurbitina* than on *C. chalybea*. All natural enemies were more common in nests with guarded female than in control nests both in *C. cucurbitina* and in *C. chalybea* (Fig. 6, Table S1), but differences were not always significant. In *C. cucurbitina*, significant difference was in the presence of chalcidoid wasps (binomial GLM, deviance = 10.53, df = 1, *P* = 0.0012; Table 2) and ants (binomial GLM, deviance = 11.30, df = 1, *P* = 0.0009; Table 2). Difference in usurpation frequency was not significant (binomial GLM, deviance = 1.2, df = 1, *P* = 0.2741; Table 2). On the other hand, in *C. chalybea* was significant difference only in nest usurpations (binomial GLM, deviance = 6.18, df = 1, *P* = 0.0129; Table 2), but not in the presence of chalcidoid wasps (binomial GLM, deviance = 0.57, df = 1, *P* = 0.4516; Table 2) and ants (binomial GLM, deviance = 2.17, df = 1, *P* = 0.134; Table 2).

We recorded the presence of other natural enemies; yet, their frequency was too low to be statistically assessed. The numbers of all the recorded natural enemies are shown in Table S1.

### Comparison between plugged nests and nest with removed female

We compared the mortality of brood cells between nests with removed females and natural plugged nests in *C. chalybea*, and the results showed that there was marginally significant difference (binomial GLM, deviance = 2.81, df = 1 *P* = 0.0932; Table S2). Difference in proportion of cells parasited by chalcidoid wasps was not significantly differ (binomial GLM, deviance = 1.31, df = 1, *P* = 0.2524).

## Discussion

### Role of nest guarding for the offspring survival

The guarding of a completely provisioned nest by a parent is obviously highly beneficial for the offspring because it increased the offspring survival in both species of *Ceratina* bees. The primary reasons for the destruction of unguarded nests were the different natural enemies, which were significantly more common in unguarded nests than in control nests of *C. cucurbitina*. In *C. chalybea* nests, the enemies were also more common in nests with the female removed; however, only the usurpation of the nests by other bees had a statistically significant effect. Natural enemies, which cause predation and parasitism pressure, are important drivers in the evolution of extended parental care in general (Wilson 1975). We attribute the observed differences between species to a different nest structure and a different guarding strategy in each species.

The positive effects of nest guarding on offspring survival are known in other nest‐making Hymenoptera; however, the current evidence is scarce. In a study by Coville and Griswold (1984), nests with a complete brood that were unguarded by a male of *Trypoxylon superbum* were destroyed by ants; however, the number of unguarded nests was extremely low (only two nests). The survival of the offspring was also significantly reduced after the removal of guarding female(s) in the eusocial *Megalopta genalis* (Smith et al. 2003) and in the communal *Lasioglossum hemichalceum* (Kukuk et al. 1998). The offspring survival was significantly correlated with the presence of a guarding female in the solitary populations of *Halictus rubicundus* (Eickwort et al. 1996). However, this latter study was only observational, and the absence of a female can be the consequence, not the cause, of the offspring death. This outcome is similar to the results of the low offspring survival and other tested features in the plugged *C. chalybea* nests of our study. The three published studies on *Ceratina* bees show ambiguous results. While the study of Sakagami and Maeta (1977) on Japanese *Ceratina* species showed a higher brood cell mortality in orphaned nests, the study of Rehan et al. (2009) on *C. accusator* found no effects of the female disappearance. However, only a small sample size was used in the latter study. Daly et al. (1967) observed a higher attack of chalcidoid wasps but not Ichneumonidae in orphaned nests than in guarded nests. In addition, all these studies were based on only observations from nest dissections, and they did not register the number of total destroyed nests because they were not considered as *Ceratina* nests. According to the literature, the primary reason for the failure of unguarded nests is the occurrence of ant raids (Coville and Griswold 1984; Kukuk et al. 1998; Smith et al. 2003). Likewise, ants are most likely the primary reason for the nest failure of *Ceratina* bees. Furthermore, the effect of ant assaults was probably underestimated in our data because the ants moved away after their raid and left the nest completely cleaned from debris and cell partitions, which makes their detection difficult.

Although we used nests in artificial nesting opportunities for our experiments, we assume that there are no differences with local natural nests in the general pattern and effect of enemies. Our artificial nests were in the immediate vicinity of natural nests, and bees and their enemies were not introduced. The observed enemies were those that usually attack *Ceratina* nests (Daly et al. 1967; Sakagami and Maeta 1977).

### Comparison of the guarding strategy in *C. cucurbitina* and *C. chalybea*

The strategy of nest defense is different between *C. cucurbitina* and *C. chalybea*, where guarding was obviously more important for *C. cucurbitina*. Interestingly, *C. cucurbitina* exhibited an obligate nest guarding and subsocial behavior, which consisted of crawling through the nest, as described in detailed studies of other *Ceratina* species (Rehan et al. 2010b). This behavior most likely protects the nest from various natural threats such as parasitism by chalcidoid wasps.

In contrast, *C. chalybea* females guard the nest only facultatively and choose between two alternative strategies, that is, they either guard the nest or fill the nest entrance with a plug and desert it. The guarding strategy is closely associated with nest architecture; guarded nests have an unclosed outermost cell in almost all the cases and, therefore, the mother is in physical contact with last offspring. In contrast, unguarded nests have a filling plug in almost all the cases (Table 1, Fig. 2). We exceptionally observed a few guarded nests with a filling plug; however, these nests were probably recently completed, and the females had not finished filling the plug and had not yet left the nest. We also observed a few unguarded nests with an unclosed outermost cell. Such nests were probably abandoned due to the death of the mother.

To the best of our knowledge, the described nest deserting behavior in *C. chalybea* represents the first example of an alternative guarding strategy within the genus *Ceratina*. Although unguarded nests were previously found in other species, these nests were most likely orphaned after the death of the mother (Sakagami and Maeta 1977; Rehan et al. 2009).

We never observed *C. chalybea* females crawling through nest partitions, and the larval excrements were always left in the cells. This pattern shows that females of *C. chalybea*, in contrast to other species, cannot crawl through the nest partitions and, therefore, cannot be in physical contact with the offspring (except for the outermost one). The higher offspring mortality in plugged nests than in guarded nests and the insignificant difference in offspring mortality between plugged nests and nests with a female removed suggest that females may also be effective against enemies. However, removing the guarding female did not have a significant influence on the mortality of the second outermost (closed) cell and on preventing the chalcidoid parasitation.

The guarded nests of *C. chalybea* have a relatively unique nest structure compared to those of other species. While the guarding female of *C. cucurbitina* (and other *Ceratina* species with known nest structure) sits on the nest entrance separated from the outermost offspring by a cell partition, the outermost cell in the guarded nests of *C. chalybea* is open, which enables a direct contact between the mother and the offspring in the outermost cell. However, no contact between the mother and the offspring in the internal cells is possible. For this reason, we consider *C. chalybea* as partially subsocial because of the direct contact of a female with a single offspring in a guarded nest, even though no contact occurs with internally positioned offspring or with offspring in plugged, deserted nests. In general, the offspring in the open cell suffered less mortality than offspring in the internal, closed cells. For instance, it was never parasitized with chalcidoid wasps when the guarding female was present.

In general, the guarding strategy of *C. cucurbitina* is more effective for the offspring survival; however, in the case of the absence of the female, the offspring are more vulnerable than in *C. chalybea*.

### Benefits and costs of guarding and deserting nests

The benefit of nest guarding is an apparently high offspring survival. We suppose that the benefit of deserting a nest is the possibility of founding a second nest elsewhere. We did not observe the fate of females that plugged and deserted nests. However, they most likely tried to found new nests. Females that plugged their nests finished them from mid‐June to July 2013. In this period, the frequency of new nest founding was similar as frequency of newly plugged nests (Fig. S2). Females that founded nests in July usually had damaged wings (M. Mikát, K. Černá and J. Straka, unpubl. obs.), which indicates that they had already been highly active and had probably founded a nest elsewhere. There was not any significant difference in date of nest founding between guarded and plugged nests, which shows that probably only some females deserted from their first nest and try to found the second nest.

We found that guarded nests had a significantly higher number of provisioned cells and live offspring than plugged nests (Table 1, Fig. 3). The lower number of provisioned cells in plugged nests indicates that *C. chalybea* females deserted less valuable nests with a higher probability. To terminate the investment in an unpromising offspring or clutch is an adaptive behavior known in other animal species (Olmstead and Wood 1990; Manica 2002). However, a question remains to be answered: Can a *C. chalybea* female assess the survival of her offspring and desert nests with higher offspring mortality or is the higher mortality only the result of the deserting strategy? The nonsignificant differences in offspring mortality and parasitism by chalcidoid wasps between plugged nests and nests with the removed female suggest that a higher mortality in plugged nests in comparison with guarded nests is the result rather than the cause of deserting.

### Evolution of the extent of parental care in *Ceratina* bees

Parental care typically tends to increase in complexity (Smiseth et al. 2012; Trumbo 2012). However, *Ceratina* bees are an example of a reduction in the extent of parental care throughout their evolution. Eusociality was lost in certain lineages of the genus *Ceratina* (Rehan et al. 2012) and, at least in *C. chalybea*, the extended maternal care further decreased by the reduction of the nest guarding activities and the loss of the ability to crawl through the cell partitions to inspect the brood.

## Data Accessibility

All the primary data were uploaded and are available as online supporting information.

## Conflict of Interest

None declared.

## Supporting information


**Figure S1.** Installing of artificial nesting opportunities in the Havraníky heathland (A).
**Figure S2.** Phenology of nest founding and nest plugging in *C. chalybea* in season 2013.
**Table S1.** Recorded natural enemies of *Ceratina* bees in nests with removed female and in control nests.
**Table S2.** Results of binomial GLM models comparing nests with removed females and plugged nests.
