# Supplementary material for: Major benefits of guarding behavior in subsocial bees: implications for social evolution
Source: Ecol Evol. 2016 Sep 1;6(19):6784–97. doi: 10.1002/ece3.2387 (PMC5513229; doi:10.1002/ece3.2387)
Supplement: Supplementary file 1 — Figure S1. Installing of artificial nesting opportunities in the Havraníky heathland (A). Figure S2. Phenology of nest founding and nest plugging in C. chalybea in season 2013. Table S1. Recorded natural enemies of Ceratina bees in nests with removed female and in control nests. Table S2. Results of binomial GLM models comparing nests with removed females and plugged nests. [file ECE3-6-6784-s001.docx]

Guarding behaviour in subsocial bees can provide major benefits and has implications for social evolution; M. Mikát, K. Černá and J. Straka; Department of Zoology, Faculty of Science, Charles University in Prague, Praha, Czech Republic e-mail: jakub.straka@aculeataresearch.com

Figure S1: Installing of artificial nesting opportunities in the Havraníky heathland (A). Sheaf of twenty *Solidago* twigs with some *Ceratina* nests (B).


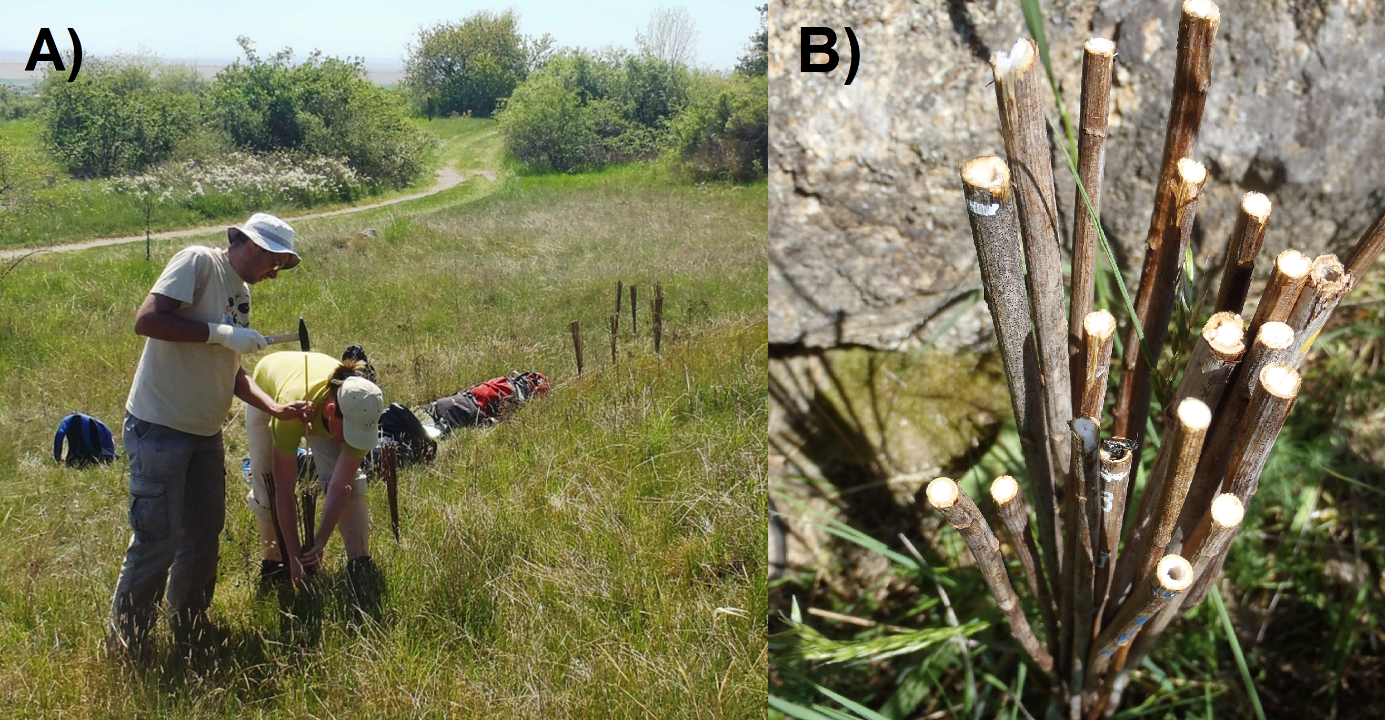


Figure S2: Phenology of nest founding and nest plugging in *C. chalybea* in season 2013.


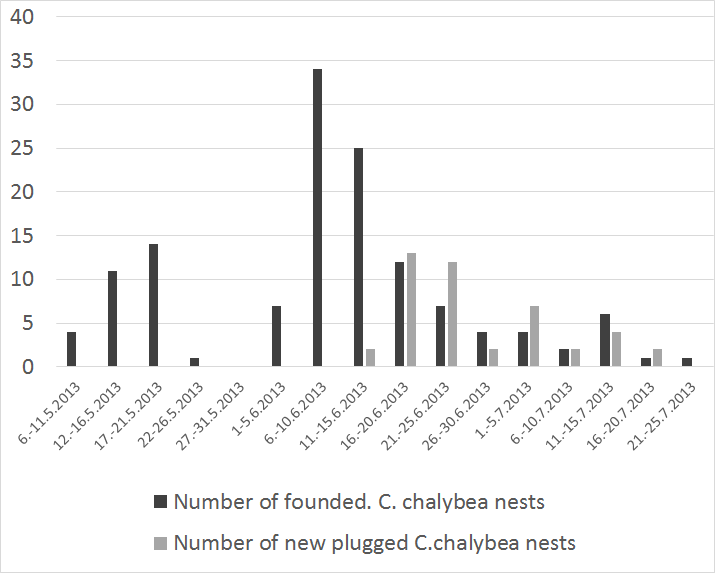


Table S1: Recorded natural enemies of *Ceratina* bees in nests with removed female and in control nests. There are shown number of nests, in which natural enemy was recorded. There was recorded more than one types of natural enemies in some nests.

| species | *C. cucurbitina* | | | | *C. chalybea* | | | |
| --- | --- | --- | --- | --- | --- | --- | --- | --- |
| treatment | control | removing | control | removing | control | removing | control | removing |
| Year | 2013 | 2013 | 2014 | 2014 | 2013 | 2013 | 2014 | 2014 |
| Number of nests | 62 | 122 | 46 | 85 | 35 | 72 | 37 | 68 |
|  |  |  |  |  |  |  |  |  |
| N of nest with live offspring | 54 | 65 | 42 | 32 | 34 | 50 | 35 | 52 |
| N of nests with all offspring died | 8 | 57 | 4 | 53 | 1 | 22 | 2 | 16 |
|  |  |  |  |  |  |  |  |  |
| Formicidae | 1 | 20 | 0 | 10 | 1 | 4 | 2 | 9 |
|  |  |  |  |  |  |  |  |  |
| Chalcidoids | 3 | 23 | 0 | 20 | 4 | 16 | 5 | 13 |
|  |  |  |  |  |  |  |  |  |
| Bees usurpation togther | 2 | 7 | 0 | 10 | 1 | 12 | 0 | 5 |
| *C. cucurbitina* | 1 | 4 | 0 | 7 | 0 | 0 | 0 | 0 |
| *C. chalybea* | 0 | 2 | 0 | 0 | 1 | 9 | 0 | 5 |
| *C. nigrolabiata* | 1 | 1 | 0 | 3 | 0 | 0 | 0 | 0 |
| *Pseudanthidium lituratum* | 0 | 0 | 0 | 0 | 0 | 1 | 0 | 0 |
| *Hoplosmia spinulosa* | 0 | 0 | 0 | 0 | 0 | 2 | 0 | 0 |
|  |  |  |  |  |  |  |  |  |
| Dermaptera | 0 | 0 | 1 | 1 | 0 | 1 | 2 | 9 |
| Ichneumonidae | 0 | 0 | 0 | 0 | 0 | 1 | 0 | 0 |
| Gasteruption sp | 0 | 0 | 0 | 0 | 1 | 1 | 1 | 2 |
| Cleridae beetle | 0 | 1 | 0 | 0 | 0 | 0 | 0 | 0 |
| nest without enemy | 56 | 73 | 45 | 46 | 28 | 38 | 27 | 32 |

Table S2: Results of binomial GLM models comparing nests with removed females and plugged nests. Full models with two explanatory variables and their interaction was used. Significant p-values are in bold.

| Dependent variable: proportion of live offspring. | | | | | |
| --- | --- | --- | --- | --- | --- |
| variable | Df | Deviance | Resid.Df | Resid. Dev | p-value |
| NULL | 60 | 39.50 |  |  |  |
| time nest abandonment (days) | 1 | 3.30 | 59 | 35.71 | 0.0515 |
| treatment (removing vs plugging) | 1 | 2.81 | 58 | 32.89 | 0.0932 |
| interaction | 1 | 0.07 | 57 | 32.82 | 0.7955 |
|  |  |  |  |  |  |
| Dependent variable: proportion of cells attacked by chalcidoids. | | | | | |
| variable | Df | Deviance | Resid. Df | Resid. Dev | p-value |
| NULL | 60 | 22.19 |  |  |  |
| time nest abandonment (days) | 1 | 1.65 | 59 | 20.54 | 0.1991 |
| treatment (removing vs plugging) | 1 | 1.31 | 58 | 19.23 | 0.2524 |
| interaction | 1 | 0.02 | 57 | 19.21 | 0.8786 |
